# Nanoscale Phase Evolution, Substitution Mechanism, and Aqueous Durability of CaZr_1−*x*_Gd_*x*_Ti_2−*x*_Nb_*x*_O_7_ (*x* = 0.1–1.0) Defect-Fluorite-Derived Ceramics

**DOI:** 10.3390/nano16110643

**Published:** 2026-05-22

**Authors:** Baolong Ma, Shixi Chen, Shiyin Ji, Chuanhang Zhao, Tian Chen

**Affiliations:** 1 Shaanxi Engineering Research Center of Advanced Nuclear Energy, School of Nuclear Science and Technology, School of Energy and Power Engineering, Xi’an Jiaotong University, Xi’an 710049, China; chenshixi@stu.xjtu.edu.cn (S.C.); 303426tianyou@stu.xjtu.edu.cn (T.C.); 2 Shaanxi Key Laboratory of Advanced Nuclear Energy and Technology, School of Nuclear Science and Technology, School of Energy and Power Engineering, Xi’an Jiaotong University, Xi’an 710049, China; 3 School of National Defense & Nuclear Science and Technology, Southwest University of Science and Technology, Mianyang 621010, China; jishy@swust.edu.cn (S.J.); zhaochuanhang1127@163.com (C.Z.)

**Keywords:** zirconolite, nanopowder, defect-fluorite-derived structure, atomic-scale substitution mechanism, aqueous durability, phase evolution

## Abstract

The safe immobilization of high-level waste (as actinide) remains a critical bottleneck in the disposal of high-level radioactive waste worldwide. Moreover, the higher specific surface area and surface energy of nano-scale powders enable the production of ceramic materials featuring denser crystal structures and superior strength, hardness, and toughness. Therefore, in this study, Gd^3+^ was used as a surrogate for actinides, and Nb^5+^ was introduced as a high-valence charge-compensating cation. Nano-scale powders of CaCO_3_, ZrO_2_, Gd_2_O_3_, TiO_2_, and Nb_2_O_5_ were employed to prepare a series of defect-fluorite-derived ceramics, CaZr_1-*x*_Gd*_x_*Ti_2-*x*_Nb*_x_*O_7_ (*x* = 0.1–1.0), via a high-temperature solid-state reaction method, aiming to investigate the atomic substitution mechanisms, phase evolution, and chemical stability under high-valence charge compensation. Laboratory X-ray diffraction (XRD), synchrotron X-ray diffraction (SXRD), and backscattered scanning electron microscopy with energy-dispersive X-ray spectroscopy (BSEM-EDX) confirmed a phase evolution sequence from zirconolite-2M to zirconolite-4M and finally to pyrochlore. This behavior is consistent with that reported for other Ln^3+^-Nb^5+^ co-doped zirconolite systems. Rietveld refinement of the SXRD data further revealed, for the first time, the site-occupancy mechanism of Gd and Nb in zirconolite-4M. In both zirconolite-2M and zirconolite-4M, Gd preferentially occupies the Ca sites, whereas Nb substitutes at the Ti sites. In the pyrochlore structure, Ca, Zr, and Gd occupy the 16d sites, while Ti and Nb occupy the 16c sites. Static leaching tests following the MCC-1 protocol showed that pyrochlore exhibits the highest leaching resistance, whereas zirconolite-2M shows the lowest. After 28 days, the highest Gd leaching rate was 1.92(1) × 10^−5^ g m^−2^ d^−1^. These results provide new insights into actinide immobilization behavior and compositional design in zirconolite-based waste forms.

## 1. Introduction

In today’s increasingly unstable geopolitical climate, many countries are reassessing the strategic role of nuclear energy. This shift is driven by the vulnerability of fossil-fuel supply chains, their uneven geographic distribution, and the sensitivity of energy prices to international disruptions. Although front-end nuclear technologies are largely mature, the back-end treatment and disposal of high-level radioactive waste (HLW) remain unresolved. This challenge has become a major bottleneck restricting the sustainable development of nuclear power. Vitrification has been considered for the engineering management of high-level radioactive waste because of its relatively low cost, broad nuclide tolerance, and mature processing technology. However, its limited solubility for highly radioactive actinides and their tendency to crystallize undesirable “yellow phases” during operation constrain further application [1]. By contrast, ceramic waste forms immobilize radionuclides through incorporation into specific crystal lattices. Therefore, they are regarded as promising matrices for actinide-bearing waste. Among them, zirconolite and other defect-fluorite-derived ceramics have shown considerable potential.

Zirconolite, with the ideal formula CaZrTiO_7_, contains three distinct cation sublattices and thus offers flexible accommodation for actinides [2,3]. As a typical defect-fluorite-derived structure, its lattice may transform after radionuclide incorporation. Common zirconolite polytypes include zirconolite-2M (two-layer monoclinic), zirconolite-4M (four-layer monoclinic), zirconolite-3O (three-layer orthorhombic), and zirconolite-3T (three-layer triclinic) [4,5,6,7,8,9,10]. When actinide or rare-earth ions are incorporated at the Zr site or jointly at the Ca and Zr sites, transformation to pyrochlore is frequently observed [11,12,13,14,15,16]. Pyrochlore is also a typical defect-fluorite-related structure with the general formula A_2_B_2_O_7_ and a face-centered cubic lattice, in which A occupies the 16d site, B occupies the 16c site, and O resides at the 48f and 8b sites [17,18,19]. Non-radioactive rare-earth elements, such as Nd, Gd, and Ho, have been widely used as surrogates to simulate actinide substitution behavior because of their similar ionic radii and physicochemical properties [12,20,21]. For example, the ionic radii of 8-coordination Ca^2+^, Zr^4+^, Nd^3+^, Sm^3+^ and Gd^3+^ in zirconolite are 1.12 Å, 0.84 Å, 1.109 Å, 1.079 Å and 1.053 Å [12,20,21]. The ionic radii of 7-coordination Ca^2+^, Zr^4+^, Sm^3+^ and Gd^3+^ in zirconolite are 1.06 Å, 0.78 Å, 1.02 Å and 1.00 Å [20].

The ionic radii of actinide and rare-earth cations are comparable to that of Ca^2+^ in zirconolite, but these cations are usually trivalent or tetravalent. Substitution for Ca^2+^ therefore creates a charge imbalance. Early studies introduced trivalent transition metal ions such as Al^3+^ and Fe^3+^ into Ti sites to achieve charge compensation and thereby enable actinide incorporation at the Ca site. These studies showed that the introduction of trivalent charge-compensating ions effectively increased the solubility limit of actinides in zirconolite-2M, and that this limit increased with decreasing ionic radius of the rare-earth element, eventually reaching 100 at.% [4,5,6]. Moreover, only a zirconolite-2M-to-zirconolite-3O phase transition was observed as the actinide content increased. X-ray absorption near-edge structure (XANES) analysis of Ca_1−*x*_Ho*_x_*ZrTi_2−*x*_(Al, Fe)*_x_*O_7_ further showed that Ho preferentially occupied the Ca site, whereas Al and Fe entered Ti sites [4]. Later, Ji et al. pioneered co-immobilization using the high-valent ion Nb^5+^ as the charge compensator. They found a zirconolite-2M → zirconolite-4M → pyrochlore phase evolution in both Nd–Nb and Sm–Nb systems [22,23]. In the zirconolite-2M structure, Nd preferentially occupied the Zr site, whereas Nb entered the Ti1 site, consistent with the design strategy. It remains unclear, however, whether Ln–Nb-codoped zirconolite systems generally follow the same phase-evolution pathway and whether the site preference of Ln cations is universal. How the ionic radii of Ln^3+^ affect the cation-substitution mechanism in zirconolite and pyrochlore therefore remains to be clarified.

Aqueous leaching resistance is a key property for ceramic waste forms intended for long-term disposal in deep geological repositories. The leaching behavior of zirconolite-based waste forms has been widely reported. Zhang et al. found that Ca_0.85_Nd_0.15_ZrTi_1.85_A_l0.15_O_7_ exhibited a leaching rate of LR_Nd_ = 3.46 × 10^−4^ g m^−2^ d^−1^ after 42 days at 90 °C following the ASTM standard [24]. Yin et al. also reported an Nd leaching rate of 4.86 × 10^−4^ g m^−2^ d^−1^ after 42 days of MCC-1 testing at 90 °C [25]. Cai et al. investigated the Nd leaching behavior of single-phase zirconolite in acidic, neutral, and alkaline solutions by the PCT test at 90 °C and found LR_Nd_ values between 3.13 × 10^−5^ and 3.97 × 10^−5^ g m^−2^ d^−1^ [26]. For zirconolite-type CaZr_1−*x*_SmxTi_2−*x*_Nb*_x_*O_7_ (*x* = 0.1–0.3), LR_Sm_ ranges from 3.04 × 10^−5^ to 6.35 × 10^−6^ g m^−2^ d^−1^ in deionized water at 90 °C [23]. For pyrochlore-type CaZr_1−*x*_Sm*_x_*Ti_2−*x*_Nb*_x_*O_7_ (*x* = 0.4–1.0), LR_Sm_ ranges from 4.49 × 10^−6^ to 4.23 × 10^−7^ g m^−2^ d^−1^ in deionized water at 90 °C [23]. Xu et al. reported that Gd_2_Zr_2_O_7_ pyrochlore exhibited LR_Gd_ = 3.17 × 10^−5^ g m^−2^ d^−1^ after 28 days of leaching in deionized water at 90 °C [27]. In addition, Teng et al. reported that the leaching rates of rare-earth elements in an A_2_Zr_2_O_7_ high-entropy pyrochlore were on the order of 10^−5^ g m^−2^ d^−1^ [28]. Overall, the leaching rates of zirconolite and pyrochlore waste forms usually fall within the range of 10^−4^ to 10^−7^ g m^−2^ d^−1^, whereas Ln–Nb-codoped zirconolite ceramics tend to show relatively lower values.

The ionic radius of Gd^3+^ (1.053 Å) is closer to that of Am^3+^ (1.09 Å) than to that of Ce^3+^ (1.143 Å) [20]. In this work, Gd^3+^ was selected as a non-radioactive surrogate for actinides, and Nb^5+^ was used as the charge-compensating ion. Nanoscale oxide powders with an average particle size of 50–100 nm were employed to prepare a series of defect-fluorite-derived ceramics, CaZr_1−*x*_Gd*_x_*Ti_2−*x*_Nb*_x_*O_7_ (*x* = 0.1–1.0), via a conventional high-temperature solid-state route. Laboratory powder X-ray diffraction (PXRD), synchrotron X-ray diffraction (SXRD), scanning electron microscopy coupled with energy-dispersive X-ray spectroscopy (SEM–EDX), and Rietveld refinement were employed to investigate the phase-evolution behavior of Gd–Nb-doped zirconolite and to clarify the substitution mechanisms of Gd and Nb in different crystal phases. Room-temperature MCC-1 static leaching experiments were conducted for 28 days to evaluate aqueous durability.

## 2. Experimental Procedures and Characterization

### 2.1. Sample Fabrication

A series of defect-fluorite-derived ceramics, CaZr_1−*x*_Gd*_x_*Ti_2−*x*_Nb*_x_*O_7_ (*x* = 0.1−1.0), was prepared by conventional solid-state sintering to investigate substitution behavior and chemical durability. The nanoscale powders of CaCO_3_, ZrO_2_, Gd_2_O_3_, TiO_2_, and Nb_2_O_5_ were purchased from Aladdin Biochemical Technology Co., Ltd. (Shanghai, China), all of which have an average particle size of 50–100 nm. First, the five raw powders, each with a purity higher than 99.99%, were weighted according to the target stoichiometry and homogenized using a vibration ball mill. The mixed powders were then uniaxially pressed into pellets at approximately 377 MPa. The round pellets, approximately 1 cm in diameter, were first sintered at 1400 °C for 24 h in air under ambient pressure. The sintered samples were subsequently re-ground, re-tableted and re-sintered at 1500 °C for 48 h to obtain more homogeneous, denser, and fully reacted products. The heating and cooling rates were set to 5 °C/min in a muffle furnace.

### 2.2. Characterization Methods

Powder X-ray diffraction (PXRD) data were collected using a Bruker D8 Advance diffractometer (Bruker AXS GmbH, Karlsruhe, Germany) in flat plate mode. The measurement was conducted using Cu Kα radiation (λ = 1.5418 Å) at 40 kV and 40 mA, with a LYNXEYE_XE_T detector (manufactured by Bruker AXS GmbH, Karlsruhe, Germany). Data were recorded over a 2θ range of 5–80° with a step size of 0.02° and a dwell time of 0.2 s/step. To obtain high-quality diffraction data for quantitative analysis, synchrotron X-ray diffraction (SXRD) measurements were performed at beamline BL 14B1 (λ = 0.6887 Å) of the Shanghai Synchrotron Radiation Facility (SSRF) [29] in capillary mode using capillaries with a diameter of 0.5 mm. The SXRD patterns were recorded by a 1D position-sensitive detector, Mythen 1k (DECTRIS Ltd., Baden, Switzerland). These patterns were collected over a 2θ range of 1–61°, with a dwell time of 20–30 s per sample. Lattice parameters and structural information were refined by Pawley and Rietveld analyses of the SXRD pattern using GSAS II [30,31,32].

Scanning electron microscope (SEM) and energy-dispersive X-ray spectroscopy (EDX) (manufactured by Carl Zeiss AG, Oberkochen, Germany) were employed to further confirm the phase distribution and to obtain semi-quantitative elemental compositions. The ceramic samples were first polished to a mirror finish using diamond pastes with particle sizes of 9, 6, 3, 1, 0.5, and 0.25 μm. The polished samples were then ultrasonically cleaned and allowed to dry naturally in air. Afterward, the sample surfaces were coated with a thin Au film. Backscattered scanning electron microscope (BSEM) and EDX characterizations were finally performed on a ZEISS Sigma 300 microscope (ZEISS, Oberkochen, Germany) equipped with an OXFORD X-MAXN20 EDX system (on the same microscope). The accelerating voltage was set to 20 kV. The EDX analysis employed a standardless method, and the semi-quantification model was based on the Cliff–Lorimer method for fast and accurate results, with automatic ZAF correction applied. The atomic ratios of the corresponding contrasts were collected from five or more spots.

### 2.3. Aqueous Leaching Experiment

The aqueous durability of the CaZr_1−*x*_Gd*_x_*Ti_2−*x*_Nb*_x_*O_7_ (*x* = 0.1–1.0) ceramic waste forms was evaluated using the static leaching protocol specified (MCC-1) in ASTM C1220 at room temperature. The unpolished pellets were immersed in 100 mL of deionized water (pH = 7) within an autoclave consisting of a polytetrafluoroethylene (PTFE) sleeve and a stainless-steel pressure vessel. The sample surface-area-to-solution-volume ratio was set to 10 cm^−1^. The normalized elemental mass losses were determined from the elemental concentrations measured in leachates collected after 1, 3, 7, 14, 21 and 28 days. The leached elemental concentrations were measured by inductively coupled plasma mass spectrometry (ICP-MS; Agilent 7700x, Agilent Technologies Inc., Santa Clara, CA, USA). The normalized leaching rates (LR) of Ca, Zr, Gd, Nb, and Ti were calculated according to Equation (1).

(1) $$LR_{i}=\frac{C_{i}V}{Sf_{i}△t}$$

Herein, *LR_i_* is the normalized leaching rate of element *i* (g∙cm^−2^∙d^−1^), *C_i_* is the concentration of element *i* (g∙cm^−3^), V is the volume of the leaching solution (cm^3^), S is the surface area of the sample (cm^2^), *f_i_* is the mass fraction of element *i* in the sample, and ∆*t* is the leaching time interval (days).

## 3. Results

### 3.1. Phase Evolution and Solubility

**Figure 1:**
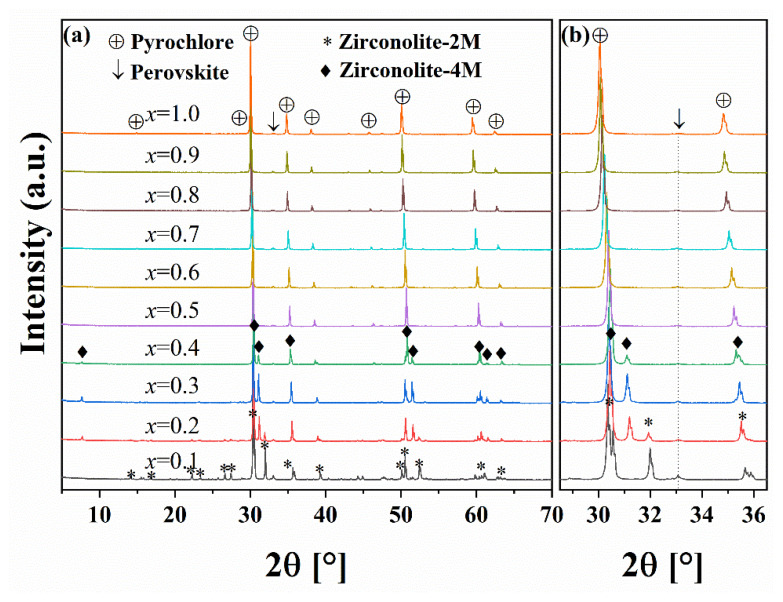
PXRD patterns of CaZr_1−*x*_Gd*_x_*Ti_2−*x*_Nb*_x_*O_7_ (*x* = 0.1−1.0) samples (**a**), and the enlarged patterns at 2θ range of 29.3–36.3° (**b**). The standard ICDD PDF numbers for the identified phases are: zirconolite-2M (PDF#84-0163), zirconolite-4M (PDF#88-0414), perovskite (PDF#82-0228), and pyrochlore (PDF#42-0002).

Figure 1 shows the PXRD patterns of CaZr_1−*x*_Gd*_x_*Ti_2−*x*_Nb*_x_*O_7_ (*x* = 0.1−1.0). For the *x* = 0.1 sample, all diffraction peaks can be indexed to zirconolite-2M (asterisk marked) and perovskite (downward arrow marked) (Figure 1a,b), indicating that the ceramic sample consists of these two phases [3,12,23]. For the *x* = 0.2 sample, two new peaks appear at 2θ = 7.7° and 31.2° (diamond marked), which are characteristic peaks of zirconolite-4M [10,23]. In addition, the characteristic peak of zirconolite-2M (about 31.9°) decreased markedly. This result indicates that the zirconolite-2M → zirconolite-4M phase transition occurred at *x* = 0.2. On the basis of the peak intensities proportion, zirconolite-4M appears to be the principal phase in the *x* = 0.2 sample. For the *x* = 0.3 sample, all diffraction peaks could be assigned to zirconolite-4M and perovskite, and no peaks of zirconolite-2M were detected (Figure 1b). The characteristic peak of zirconolite-4M (about 31.2°) increased continuously and became the dominant phase. The XRD pattern of the *x* = 0.4 sample can also be indexed to zirconolite-4M and perovskite. However, the diffraction peak intensities of zirconolite-4M (as 31.2°) are markedly weaker than those of the *x* = 0.3 sample, suggesting the generation of a new crystal phase in the *x* = 0.4 sample. This phase is most likely pyrochlore, because the major peaks of zirconolite-4M and pyrochlore overlap and the pyrochlore characteristic peak near 50.8° becomes more pronounced. For *x* = 0.5–1.0 samples, the diffraction peaks can be assigned to the pyrochlore phase [12,28,33]. Additionally, a very weak peak of perovskite was observed at near 33.1°. Therefore, the phase evolution of zirconolite-2M to zirconolite-4M, and then to pyrochlore was identified by XRD results. The phase evolution is consistent with nanoscale cation substitution progressively destabilizing the 2M structure in favor of 4M and finally pyrochlore.

**Figure 2:**
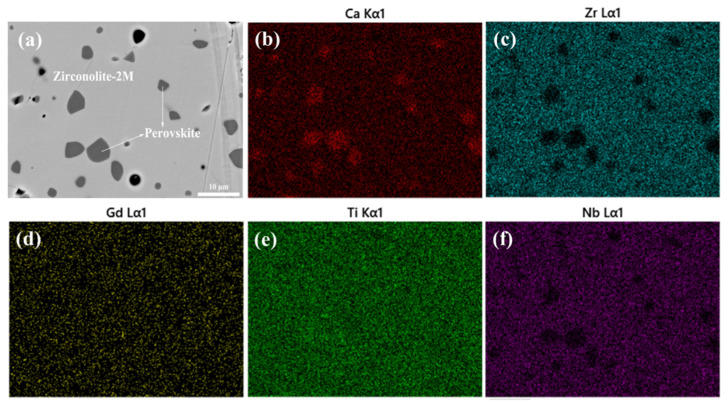
BSEM image (**a**) and corresponding EDX mapping results (**b**–**f**) for the *x* = 0.1 sample.

Figure 2 shows the BSEM image and corresponding elemental maps of the *x* = 0.1 sample. Two contrast regions are visible in the BSEM image (Figure 2a). Combined with the XRD results and the compositional criteria obtained from BSEM analysis, the gray region can be assigned to zirconolite-2M, whereas the dark-gray region corresponds to perovskite. The elemental maps further show Ca enrichment and depletion of Zr and Nb in the dark-gray region, confirming that this region is the perovskite impurity phase (Figure 2b–f).

**Figure 3:**
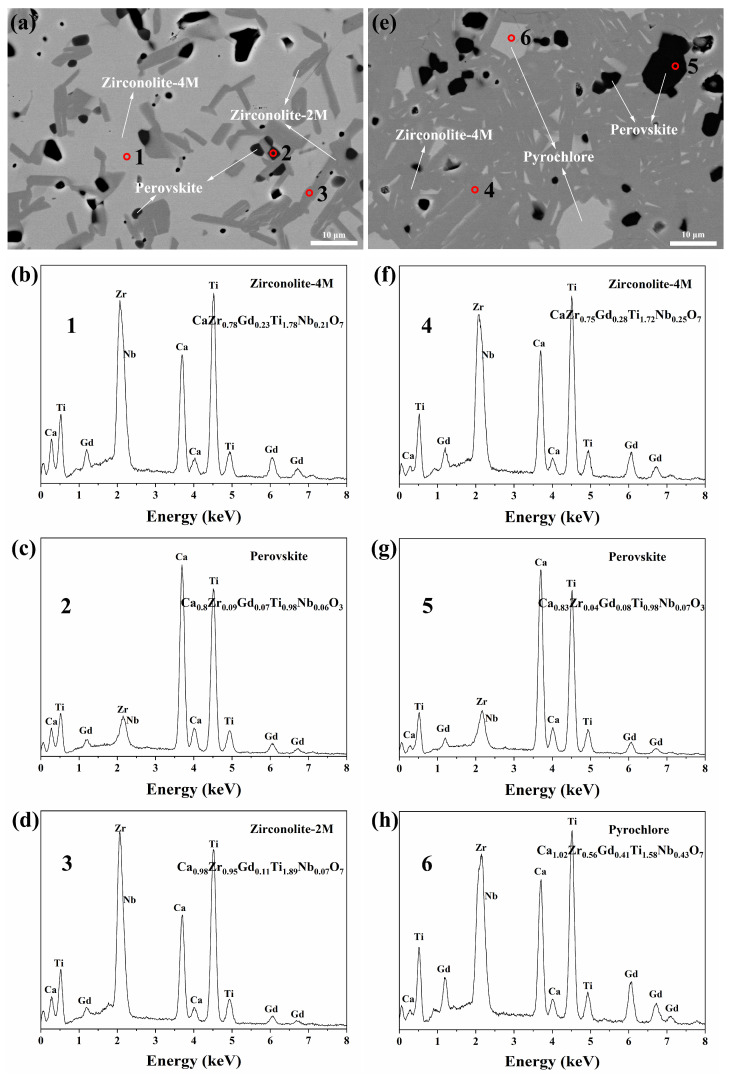
BSEM images and corresponding EDX spectra of CaZr_1−*x*_Gd*_x_*Ti_2−*x*_Nb*_x_*O_7_ samples: (**a**–**d**) *x* = 0.2; (**e**–**h**) *x* = 0.3.

Figure 3 presents the BSEM images and EDX results of the *x* = 0.2 and *x* = 0.3 samples. In the *x* = 0.2 sample, three contrast regions are observed in the BSEM image (Figure 3a). Combined with the XRD and EDX results, these regions can be identified as zirconolite-2M (dark gray), zirconolite-4M (gray), and perovskite (black), respectively. Their semi-quantitative compositions are Ca_0.98_Zr_0.95_Gd_0.11_Ti_1.89_Nb_0.07_O_7_ for zirconolite-2M, Ca_1.00_Zr_0.78_Gd_0.23_Ti_1.78_Nb_0.21_O_7_ for zirconolite-4M, and Ca_0.80_Zr_0.09_Gd_0.07_Ti_0.98_Nb_0.06_O_3_ for perovskite, as shown in Figure 3b–d. The BSEM image of the *x* = 0.3 sample likewise shows gray, dark-gray, and black regions (Figure 3e). Based on the EDX results, the black region is identified as perovskite (Figure 3g). The semi-quantitative elemental compositions of the gray and dark-gray regions are Ca_1.02_Zr_0.56_Gd_0.41_Ti_1.58_Nb_0.43_O_7_ and Ca_1.00_Zr_0.75_Gd_0.28_Ti_1.72_Nb_0.25_O_7_, respectively (Figure 3f,h). Combined with the XRD result showing zirconolite-4M as the major phase, the dark-gray region is assigned to zirconolite-4M. The gray region contains markedly higher Gd and Nb contents than the dark-gray region and is therefore attributed to pyrochlore. No obvious characteristic pyrochlore peaks are resolved in the XRD pattern, probably because they are masked by the stronger zirconolite-4M reflections; similar behavior has been reported previously [22,23]. Therefore, the combined XRD and BSEM–EDX results indicate that the zirconolite-2M → zirconolite-4M and zirconolite-4M → pyrochlore transitions occur at *x* = 0.2 and *x* = 0.3, respectively. Similar phase evolution has also been reported previously [13,14,34].

**Figure 4:**
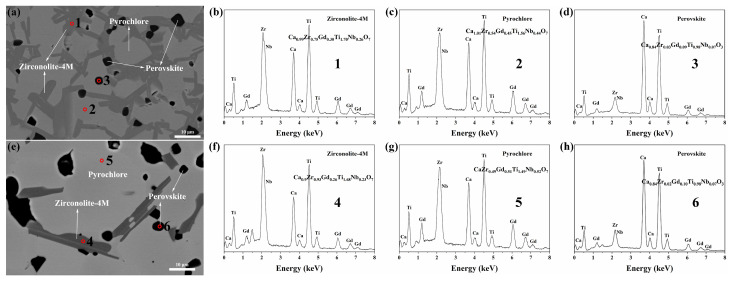
BSEM images and corresponding EDX spectra of CaZr_1−*x*_Gd*_x_*Ti_2−*x*_Nb*_x_*O_7_ samples: (**a**–**d**) *x* = 0.4; (**e**–**h**) *x* = 0.5.

Figure 4 shows the BSEM–EDX results for the *x* = 0.4 and *x* = 0.5 samples. In the *x* = 0.4 sample, three contrast regions are present in the BSEM image (Figure 4a), corresponding to pyrochlore (light gray), zirconolite-4M (gray), and perovskite (dark), consistent with the XRD results shown in Figure 1. The semi-quantitative compositions determined by EDX are Ca_0.99_Zr_0.75_Gd_0.30_Ti_1.70_Nb_0.26_O_7_ for zirconolite-4M, Ca_1.01_Zr_0.54_Gd_0.45_Ti_1.56_Nb_0.44_O_7_ for pyrochlore, and Ca_0.84_Zr_0.03_Gd_0.09_Ti_0.98_Nb_0.07_O_3_ for perovskite (Figure 4b–d). Three contrast levels are also observed in the *x* = 0.5 sample. Based on the XRD and EDX results (Figure 4g,h), the gray and black regions can be assigned to pyrochlore and perovskite, respectively. According to the phase-evolution sequence, the dark-gray region is most likely zirconolite-4M. However, no characteristic zirconolite-4M reflection is observed in the XRD pattern, probably because its content is too low to exceed the XRD detection limit. The semi-quantitative EDX result further supports this assignment (Figure 4f).

**Figure 5:**
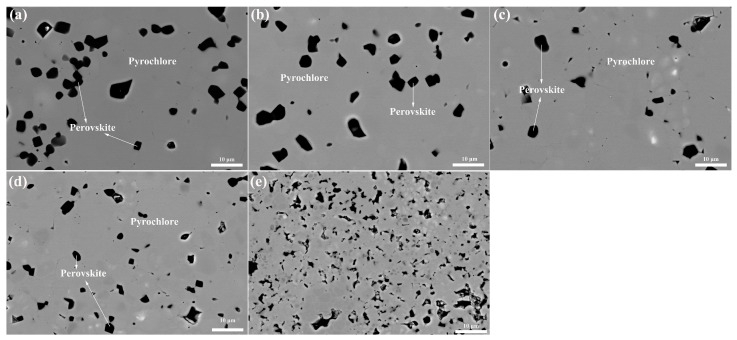
BSEM images of CaZr_1−*x*_Gd*_x_*Ti_2−*x*_Nb*_x_*O_7_ samples: (**a**) *x* = 0.6; (**b**) *x* = 0.7; (**c**) *x* = 0.8; (**d**) *x* = 0.9; (**e**) *x* = 1.0.

**Figure 6:**
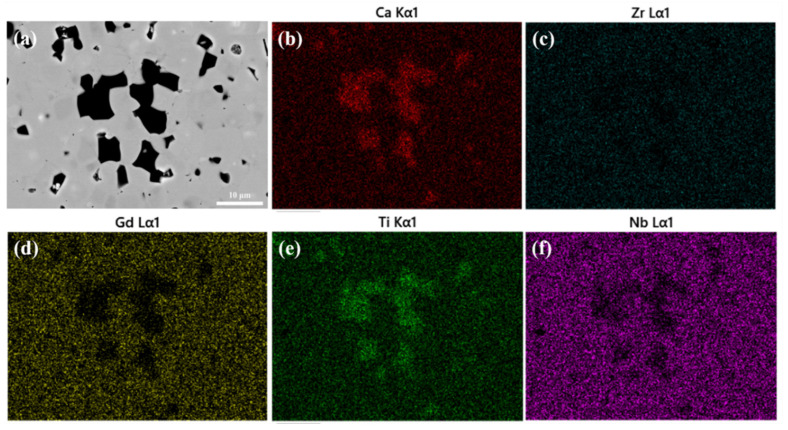
BSEM image (**a**) and corresponding mapping results (**b**–**f**) of CaZr_1−*x*_Gd*_x_*Ti_2−*x*_Nb*_x_*O_7_ (*x* = 0.9) samples.

**Figure 7:**
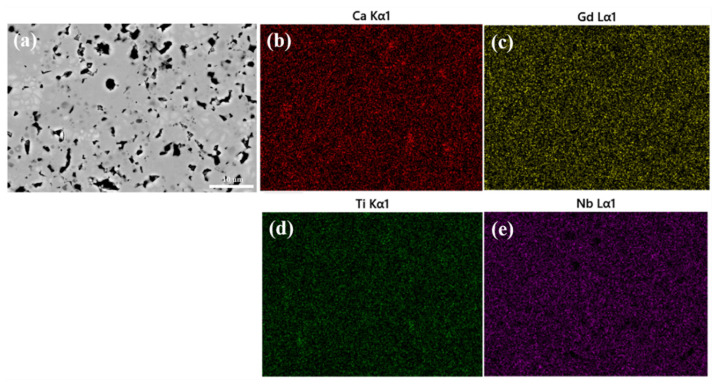
BSEM image (**a**) and corresponding mapping results (**b**–**e**) of CaZr_1−*x*_Gd*_x_*Ti_2−*x*_Nb*_x_*O_7_ (*x* = 1.0) samples.

Figure 5 shows the BSEM images of the *x* = 0.6–1.0 samples. In all cases, two contrast regions are observed and can be assigned to pyrochlore (gray) and perovskite (black), respectively. Bright spots are also visible in the BSEM images of the *x* = 0.8 and *x* = 0.9 samples (Figure 5c,d). To clarify the nature of these bright spots, elemental mapping was carried out, and the results are shown in Figure 6. The black region is confirmed to be Ca- and Ti-rich perovskite, whereas the bright spots show no obvious compositional difference from the gray region. This result indicates that no new phase forms at the bright spots, in agreement with the XRD results. Magnified BSEM images further show that the bright spots are mainly located near grain boundaries of fine grains, suggesting that they are caused by local charge accumulation. In addition, the mapping results of the *x* = 1.0 sample show that this sample is relatively less dense and contains obvious small pores (Figure 7).

To further evaluate the phase-evolution pathway and solid solubility upon Gd–Nb substitution on a semi-quantitative basis, the EDX-derived elemental compositions of CaZr_1−*x*_Gd*_x_*Ti_2−*x*_Nb*_x_*O_7_ (*x* = 0.1–1.0) are summarized in Table 1. The results confirm a zirconolite-2M → zirconolite-4M → pyrochlore phase-evolution sequence, with phase-transition points at x = 0.2 and x = 0.3. The Gd solubility in zirconolite-2M is approximately 11 at.%. The Gd solid-solution ranges in zirconolite-4M and pyrochlore are 23–30 at.% and 41–95 at.%, respectively. By contrast, the perovskite impurity phase shows a relatively low and nearly constant Gd solubility of about 6–10 at.%.

### 3.2. Substitution Mechanism in Defect-Fluorite-Derived Ceramics

**Figure 8:**
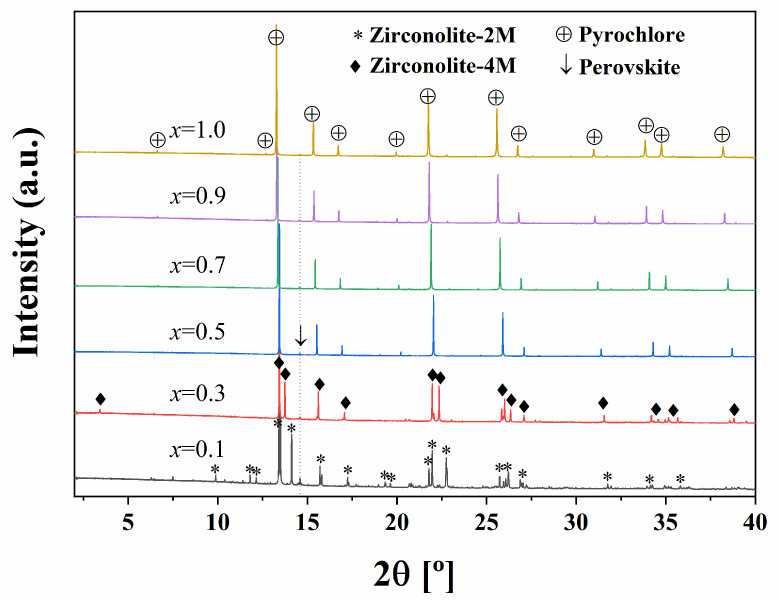
SXRD patterns of CaZr_1−*x*_Gd*_x_*Ti_2−*x*_Nb*_x_*O_7_ (*x* = 0.1–1.0) samples.

To further elucidate the site occupancy of Gd and Nb in defect-fluorite-derived ceramics, SXRD measurements were performed on CaZr_1−*x*_Gd*_x_*Ti_2−*x*_Nb*_x_*O_7_ samples with *x* = 0.1, 0.3, 0.5, 0.7, 0.9, and 1.0. The results are shown in Figure 8. If the influence of the trace perovskite impurity is ignored, the *x* = 0.1 and *x* = 0.3 samples can be regarded as single-phase zirconolite-2M and zirconolite-4M, respectively, whereas the *x* = 0.5–1.0 samples are approximately single-phase pyrochlore. This conclusion is consistent with the laboratory PXRD results (Figure 1). The characteristic reflections of trace pyrochlore in the *x* = 0.3 sample and trace zirconolite-4M in the *x* = 0.5 sample are not resolved in the SXRD patterns, indicating that their contents are too low and their diffraction intensities are masked by the dominant phase. These trace phases were therefore neglected in the subsequent refinement analysis.

**Figure 9:**
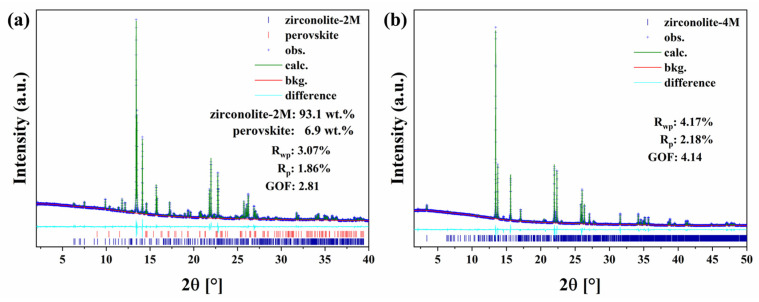
Rietveld-refined SXRD patterns of CaZr_1−*x*_Gd*_x_*Ti_2−*x*_Nb*_x_*O_7_ samples: (**a**) *x* = 0.1; (**b**) *x* = 0.3.

Figure 9a shows the Rietveld refinement of the SXRD pattern for the *x* = 0.1 sample. The initial zirconolite-2M and perovskite structures are from Ji, Whittle and Liu et al. [3,22,35]. The calculated pattern agrees well with the observed data, with residual factors of R_wp_ = 3.07%, R_p_ = 1.86%, GOF = 2.81. An R_wp_ value below 10% is generally considered indicative of an acceptable fit and the values obtained here fall within this range, confirming that the structural models are consistent with the experimental data [36,37]. The refined phase fraction of zirconolite-2M is about 93.1 wt.%, and the refined composition was determined to be Ca_1.00(1)_Zr_0.95(2)_Gd_0.05(1)_Ti_1.91(1)_Nb_0.09(1)_O_7_, which is close to the semi-quantitative EDX result. The refined structural parameters of zirconolite-2M are listed in Table 2. Gd preferentially occupies the Ca site, whereas Nb occupies the Ti1 site. This site preference differs from that reported for the Nd–Nb-doped zirconolite system, probably because of the different ionic radii of Gd and Nd [20,22]. This is the first report of the atomic-scale substitution mechanism in Gd–Nb-doped zirconolite-2M. The ionic radii of Gd^3+^ and Nd^3+^ are 1.053 and 1.109 Å in eightfold coordination, and 1.000 and 1.020 Å in sevenfold coordination, respectively [20]. By comparison, the ionic radii of Ca^2+^ and Zr^4+^ are 1.12 and 0.84 Å in eightfold coordination, and 1.06 and 0.78 Å in sevenfold coordination, respectively [20,22]. Therefore, occupation of the Ca site by Gd is expected to induce less structural distortion in zirconolite-2M.

Figure 9b presents the Rietveld refinement of the SXRD pattern for the *x* = 0.3 sample. Because the perovskite content is low, it was not included in the refinement, and only zirconolite-4M was refined. The initial structural model of zirconolite-4M was taken from the report of Coelho et al. [10]. The refined structural information for zirconolite-4M is summarized in Table 3, and the refined lattice parameters are *a* = 12.4100(14) Å, *b* = 7.1598(7) Å, *c* = 22.8954(27) Å, and *β* = 84.834(2)°. The refined composition of zirconolite-4M should be Ca_1.08(1)_Zr_0.67(1)_Gd_0.25(1)_Ti_1.75(1)_Nb_0.25(1)_O_7_, which agrees well with the semi-quantitative EDX result. In this structure, Gd preferentially occupies Ca sites, with the highest occupancy at the Ca3 position. Nb is distributed over the Ti1, Ti3, and Ti4 sites. The crystal-structure information of Gd–Nd substituted zirconolite-4M is reported here for the first time.

**Figure 10:**
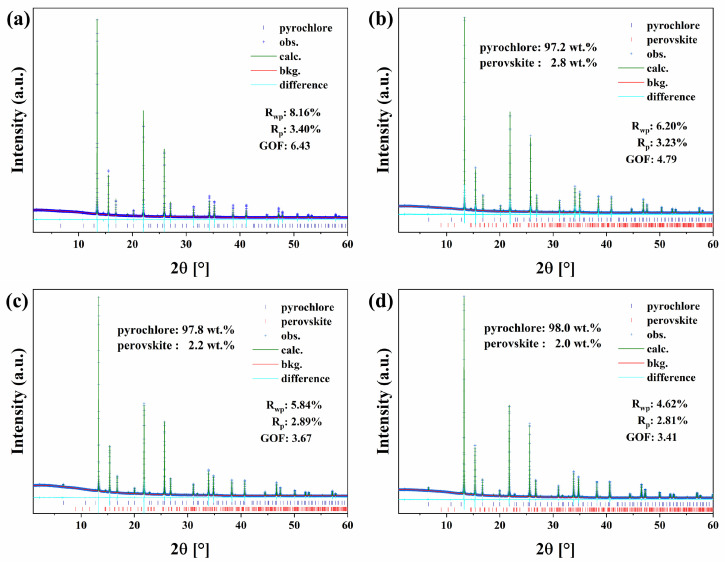
Rietveld-refined SXRD patterns of CaZr_1−*x*_Gd*_x_*Ti_2−*x*_Nb*_x_*O_7_ samples: (**a**) *x* = 0.5; (**b**) *x* = 0.7; (**c**) *x* = 0.9; (**d**) *x* = 1.0.

The Rietveld refinements of the SXRD patterns for the *x* = 0.5, 0.7, 0.9, and 1.0 samples are shown in Figure 10. The initial structural model of pyrochlore was taken from Istomin [38]. Perovskite was included as a trace phase in the refinement, although its atomic occupancies were not refined. The *x* = 0.5 sample may contain an extremely small amount of zirconolite-4M, but no clearly distinguishable diffraction peaks are observed. Because neither perovskite nor zirconolite-4M was refined for *x* = 0.5, the residual value R_wp_ = 8.16% is slightly higher than those of the other three samples. However, the calculated patterns fit the experimental data well and satisfy commonly accepted criteria for Rietveld refinement, confirming that the synthesized ceramics are essentially near-single-phase pyrochlore [36,37]. As *x* increases, the pyrochlore phase fraction rises slightly, from 97.2 wt.% for *x* = 0.7 to 98.0 wt.% for *x* = 1.0. The refined lattice parameters, x coordinate of the 48f oxygen site and cation occupancies of pyrochlore are listed in Table 4. The lattice parameter increases with increasing *x* as listed in Table 4. In the pyrochlore structure, Ca, Zr, and Gd occupy the 16d site, whereas Ti and Nb occupy the 16c site. This result is consistent with that of the Nd–Nb series. The nanoscale resolution of SXRD coupled with Rietveld refinement allowed unambiguous determination of site occupancies, demonstrating that the substitution mechanism is inherently nanoscale, as it involves individual cation sites within the defect-fluorite-derived lattice.

### 3.3. Chemical Durability

**Figure 11:**
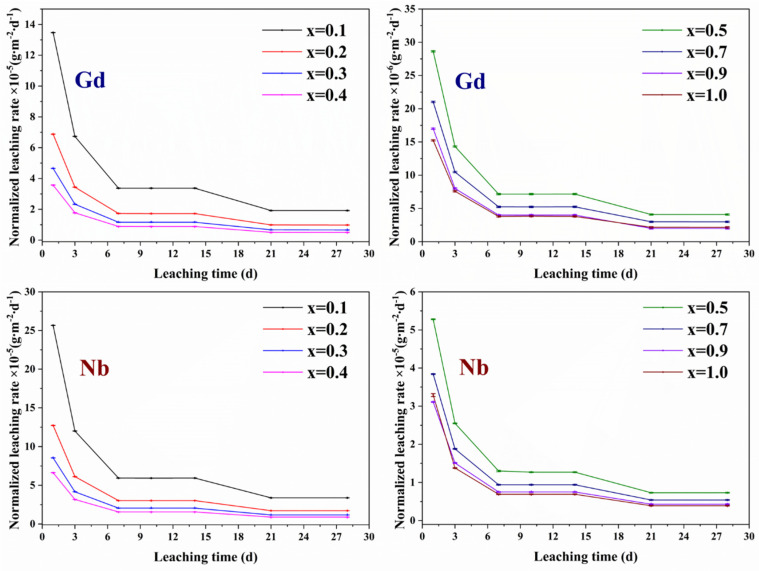
Normalized leaching rates of Gd^3+^ and Nb^5+^ in CaZr_1−*x*_Gd*_x_*Ti_2−*x*_Nb*_x_*O_7_ (*x* = 0.1−0.5, 0.7, 0.9, 1.0) samples.

The leaching rate of radionuclides from high-level radioactive waste forms is a key indicator for evaluating long-term disposal performance. In this study, room-temperature leaching experiments were carried out on CaZr_1−*x*_Gd*_x_*Ti_2−*x*_Nb*_x_*O_7_ samples with *x* = 0.1, 0.2, 0.3, 0.4, 0.5, 0.7, 0.9, and 1.0. The leaching-rate curves of Gd and Nb are shown in Figure 11. For the *x* = 0.1–0.4 samples, the Gd leaching rate decreases with increasing *x*, and the Nb leaching curves show a similar trend. Combined with the XRD and BSEM–EDX results, and neglecting the trace perovskite impurity, the *x* = 0.1 sample can be regarded as nearly single-phase zirconolite-2M; the *x* = 0.2 sample is a mixed zirconolite-2M/zirconolite-4M ceramic; the *x* = 0.3 sample is a zirconolite-4M ceramic containing trace pyrochlore; and the *x* = 0.4 sample is a mixed pyrochlore/zirconolite-4M ceramic. These results suggest that the aqueous durability of the three phases follows the order pyrochlore > zirconolite-4M > zirconolite-2M. For pyrochlore ceramics, the leaching rates of Gd and Nb decrease as their contents increase. Notably, the Gd leaching curves of the *x* = 0.9 and *x* = 1.0 samples almost overlap. In the BSEM images, pores and the perovskite phase were observed as dark/black areas. Because of local charge accumulation, bright rings can be observed at pore boundaries rather than at perovskite phase boundaries (Figure 5). The mapping results in Figure 6 and Figure 7 further confirmed that the *x* = 1.0 sample was more porous than the *x* = 0.9 sample. A higher porosity implies a larger surface area available for leaching, resulting in a higher leaching rate. This could explain the abnormal leaching curve of the *x* = 1.0 sample: it almost overlaps with the *x* = 0.9 curve instead of lying below it. After 28 days of leaching, the Gd leaching rates of the *x* = 0.1, 0.3, 0.4, and 0.9 samples are approximately 1.92(1) × 10^−5^ g m^−2^ d^−1^, 6.65(1) × 10^−6^ g m^−2^ d^−1^, 5.05(1) × 10^−6^ g m^−2^ d^−1^, and 2.16(1) × 10^−6^ g m^−2^ d^−1^, respectively. These values are of the same order of magnitude as those reported for the Sm–Nb system [21].

## 4. Conclusions

This study systematically investigated the phase evolution, atomic-scale substitution mechanism, and aqueous durability of CaZr_1−*x*_Gd*_x_*Ti_2−*x*_Nb*_x_*O_7_ (*x* = 0.1–1.0) ceramics. Laboratory XRD, SXRD, and BSEM-EDX analyses showed that increasing Gd and Nb substitution drives a sequential phase transformation from zirconolite-2M to zirconolite-4M and finally to pyrochlore. This result indicates that zirconolite-based matrices exhibit a consistent phase-evolution pathway for high-level radionuclide immobilization under charge compensation by high-valence Nb^5+^. Rietveld refinement of the SXRD data showed that, in zirconolite-2M, Gd preferentially occupies the Ca site, whereas Nb remains at the Ti1 site. This represents a new observation for the Gd–Nb-doped zirconolite system. The study also reveals, for the first time, the atomic scale substitution mechanism of Gd and Nb in zirconolite-4M: Gd preferentially enters the Ca sites, whereas Nb occupies Ti sites. In the pyrochlore structure, Ca, Zr, and Gd occupy the 16d sites, while Ti and Nb occupy the 16c sites, consistent with the Nd–Nb series. Static MCC-1 leaching tests further showed that the leaching rates of Gd and Nb decrease progressively with increasing Gd–Nb content in the CaZr_1−*x*_Gd*_x_*Ti_2−*x*_Nb*_x_*O_7_ system. Combined with the phase assemblage analysis, the aqueous durability of the three major phases follows the order pyrochlore > zirconolite-4M > zirconolite-2M. These findings provide important nanoscale insights for compositional design in the co-immobilization of actinides using high-valence charge compensation in ceramic waste forms, and demonstrate that defect-fluorite derived ceramics synthesized from nanopowders exhibit good chemical durability.

## Figures and Tables

**Table 1:** Semi-quantitative elemental compositions of the defect-fluorite-derived phases in CaZr_1−*x*_Gd*_x_*Ti_2−*x*_Nb*_x_*O_7_ samples.

	Zirconolite-2M	Zirconolite-4M	Pyrochlore	Perovskite
*x* = 0.1	Ca_0.97_Zr_0.93_Gd_0.10_Ti_1.93_Nb_0.09_O_7_			Ca_0.86_Zr_0.04_Gd_0.06_Ti_0.99_Nb_0.05_O_3_
*x* = 0.2	Ca_0.98_Zr_0.95_Gd_0.11_Ti_1.89_Nb_0.07_O_7_	Ca_1.00_Zr_0.78_Gd_0.23_Ti_1.78_Nb_0.21_O_7_		Ca_0.80_Zr_0.09_Gd_0.07_Ti_0.98_Nb_0.06_O_3_
*x* = 0.3		Ca_1.00_Zr_0.75_Gd_0.28_Ti_1.72_Nb_0.25_O_7_	Ca_1.02_Zr_0.56_Gd_0.41_Ti_1.58_Nb_0.43_O_7_	Ca_0.83_Zr_0.04_Gd_0.08_Ti_0.98_Nb_0.07_O_3_
*x* = 0.4		C_a0.99_Zr_0.75_Gd_0.30_Ti_1.70_Nb_0.26_O_7_	Ca_1.01_Zr_0.54_Gd_0.45_Ti_1.56_Nb_0.44_O_7_	Ca_0.84_Zr_0.03_Gd_0.09_Ti_0.98_Nb_0.07_O_3_
*x* = 0.5		Ca_0.90_Zr_0.93_Gd_0.26_Ti_1.68_Nb_0.23_O_7_	Ca_1.00_Zr_0.49_Gd_0.51_Ti_1.49_Nb_0.52_O_7_	Ca_0.84_Zr_0.02_Gd_0.10_Ti_0.98_Nb_0.07_O_3_
*x* = 0.6			Ca_0.99_Zr_0.42_Gd_0.60_Ti_1.38_Nb_0.61_O_7_	Ca_0.84_Zr_0.02_Gd_0.09_Ti_0.96_Nb_0.09_O_3_
*x* = 0.7			Ca_1.00_Zr_0.33_Gd_0.68_Ti_1.28_Nb_0.71_O_7_	Ca_0.86_Zr_0.02_Gd_0.08_Ti_0.94_Nb_0.10_O_3_
*x* = 0.8			Ca_1.00_Zr_0.21_Gd_0.79_Ti_1.19_Nb_0.81_O_7_	Ca_0.86_Zr_0.01_Gd_0.07_Ti_0.93_Nb_0.12_O_3_
*x* = 0.9			Ca_0.99_Zr_0.10_Gd_0.90_Ti_1.08_Nb_0.93_O_7_	Ca_0.86_Zr_0.01_Gd_0.07_Ti_0.94_Nb_0.12_O_7_
*x* = 1.0			Ca_0.99_Gd_1.00_Ti_0.95_Nb_1.07_O_7_	Ca_0.90_Gd_0.07_Ti_0.94_Nb_0.09_O_3_

**Table 2:** Rietveld-refined structural information of zirconolite-2M in CaZr_1−*x*_Gd*_x_*Ti_2−*x*_Nb*_x_*O_7_ (*x* = 0.1) sample.

Atom	Wyc.	*x*	*y*	*z*	Occ.	Uiso.
Ca1	*8f*	0.3717(3)	0.1235(6)	0.4931(3)	0.95(1)	0.008(2)
Gd1	*8f*	0.3717(3)	0.1235(6)	0.4931(3)	0.05(1)	0.008(2)
Ca2	*8f*	0.1210(2)	0.1208(5)	0.9770(2)	0.05(2)	0.019(3)
Zr1	*8f*	0.1210(2)	0.1208(5)	0.9770(2)	0.95(2)	0.019(3)
Ti1	*8f*	0.2475(4)	0.1224(9)	0.7434(4)	0.91(1)	0.021(3)
Nb1	*8f*	0.2475(4)	0.1224(9)	0.7434(4)	0.09(1)	0.021(3)
Ti2	*8f*	0.3978(9)	−0.1414(20)	0.2128(8)	0.50	0.085(5)
Ti3	*4e*	0.5	0.0670(11)	0.25	1.00	0.080(4)
O1	*8f*	0.3023(8)	0.1175(2)	0.2853(8)	1.00	0.047(5)
O2	*8f*	0.4655(7)	0.1280(19)	0.0946(7)	1.00	0.009(4)
O3	*8f*	0.2039(12)	0.1034(23)	0.5614(10)	1.00	0.075(7)
O4	*8f*	0.500(45)	0.3600(14)	0.749(38)	1.00	0.007(4)
O5	*8f*	0.7135(10)	0.1884(13)	0.5850(8)	1.00	0.023(5)
O6	*8f*	−0.0124(7)	0.1199(20)	0.4156(8)	1.00	0.379(5)
O7	*8f*	0.1153(9)	0.0434(13)	0.7946(9)	1.00	0.020(5)

Monoclinic, space group: C 1 2/c 1 (No. 15), phase fraction: 93.1 wt.%. Refined composition: Ca_1.00(1)_Zr_0.95(2)_Gd_0.05(1)_Ti_1.91(1)_Nb_0.09(1)_O_7_, a = 12.323(2) Å; b = 7.191(1) Å; c = 11.241(2) Å; β = 100.589(4)°; V = 979.1(4) Å^3^. R_wp_ = 3.07%; R_p_ = 1.86%; GOF = 2.81.

**Table 3:** The Rietveld-refined structural information of zirconolite-4M in the CaZr_1-*x*_Gd*_x_*Ti_2-*x*_Nb*_x_*O_7_ (*x* = 0.3) sample.

Atom	Wyc.	*x*	*y*	*z*	Occ.	Uiso.
Ca1	4e	0	0.1305(32)	0.25	0.83(2)	0.009(6)
Gd1	4e	0	0.1305(32)	0.25	0.17(2)	0.009(6)
Ca2	8f	0.7505(16)	0.8849(28)	0.2546(6)	0.84(1)	0.182(2)
Gd2	8f	0.7505(16)	0.8849(28)	0.2546(6)	0.16(1)	0.182(2)
Ca3	8f	0.8772(11)	0.3717(25)	0.5034(5)	0.74(2)	0.019(3)
Gd3	8f	0.8772(11)	0.3717(25)	0.5034(5)	0.26(2)	0.019(3)
Zr1	4e	0	0.6336(32)	0.25	0.67(9)	0.003(5)
Ca4	4e	0	0.6336(32)	0.25	0.33(9)	0.003(5)
Zr2	8f	0.8807(16)	0.8801(33)	0.4994(6)	1	0.044(6)
Ti1	8f	0.0637(17)	0.8764(35)	0.3745(7)	0.86(6)	0.023(7)
Nb1	8f	0.0637(17)	0.8764(35)	0.3745(7)	0.14(6)	0.023(7)
Ti2	8f	0.8145(42)	0.1403(70)	0.3906(13)	0.5	0.007(9)
Ti3	8f	0.4319(18)	0.1031(31)	0.6263(7)	0.87(5)	0.029(9)
Nb2	8f	0.4319(18)	0.1031(31)	0.6263(7)	0.13(5)	0.029(9)
Ti4	8f	0.1890(35)	0.8680(63)	0.6244(12)	0.27(4)	0.009(7)
Nb3	8f	0.1890(35)	0.8680(63)	0.6244(12)	0.23(4)	0.009(7)
Ti5	8f	0.8200(18)	0.6298(31)	0.3784(8)	1	0.013(5)
O1	8f	−0.0069(51)	0.2055(83)	0.3600(22)	1	0.035(21)
O2	8f	0.7876(41)	0.0974(72)	0.4673(18)	1	0.003(13)
O3	8f	0.0276(46)	0.9052(70)	0.4633(20)	1	0.003(15)
O4	8f	0.9075(61)	0.4306(80)	0.4055(24)	1	0.058(24)
O5	8f	0.0998(43)	0.9186(62)	0.2896(18)	1	0.001(14)
O6	8f	0.7733(42)	0.6622(80)	0.4568(18)	1	0.001(15)
O7	8f	0.9006(42)	0.9333(60)	0.3895(20)	1	0.002(12)
O8	8f	0.3713(63)	0.7869(99)	0.6156(24)	1	0.064(25)
O9	8f	0.1435(60)	0.8624(102)	0.7028(24)	1	0.048(24)
O10	8f	0.3873(36)	0.1172(83)	0.6989(18)	1	0.001(13)
O11	8f	0.2719(44)	0.5312(62)	0.6413(18)	1	0.001(14)
O12	8f	0.4563(44)	0.1264(81)	0.5402(19)	1	0.004(11)
O13	8f	0.1507(37)	0.3385(62)	0.6920(16)	1	0.002(13)
O14	8f	0.2780(38)	0.1503(66)	0.6417(15)	1	0.001(10)

Monoclinic, Space group: C 1 2/c 1 (No. 15), Refined composition: Ca_1.08(1)_Zr_0.67(1)_ Gd_0.25(1)_Ti_1.75(1)_Nb_0.25(1)_O_7_. a = 12.4100(14) Å; b = 7.1598(7) Å; c = 22.8954(27) Å; β = 84.834(2)°; V = 2026.1(6) Å^3^. R_wp_ = 4.17%; R_p_ = 2.18%; GOF = 4.14.

**Table 4:** Rietveld refinement results for the pyrochlore structure in CaZr_1−*x*_Gd*_x_*Ti_2−*x*_Nb*_x_*O_7_ (*x* = 0.5, 0.7, 0.9, 1.0) samples.

Sample	*a* (Å)	*x_48f_*	16c	16d	Refinement Residuals
*x* = 0.5	10.1794(1)	0.3227(8)	0.73Ti + 0.27Nb	0.54Ca + 0.20Zr + 0.26Nd	R_wp_ = 8.16%, R_p_ = 3.40%, GOF = 6.43
*x* = 0.7	10.2714(1)	0.3235(5)	0.57Ti + 0.43Nb	0.5Ca + 0.19Zr + 0.32Gd	R_wp_ = 6.20%, R_p_ = 3.23%, GOF = 4.79
*x* = 0.9	10.2878(1)	0.3221(3)	0.46Ti + 0.54Nb	0.5Ca + 0.01Zr + 0.49Gd	R_wp_ = 5.84%, R_p_ = 2.89%, GOF = 3.67
*x* = 1.0	10.3287(1)	0.3225(3)	0.41Ti + 0.59Nb	0.46Ca + 0.54Gd	R_wp_ = 4.62%, R_p_ = 2.81%, GOF = 3.41

## Data Availability

The raw data supporting the conclusions of this article will be made available by the authors on request.
